# EZH2 is a negative prognostic biomarker associated with immunosuppression in hepatocellular carcinoma

**DOI:** 10.1371/journal.pone.0242191

**Published:** 2020-11-12

**Authors:** Baoping Guo, Xiaohong Tan, Hong Cen

**Affiliations:** Department of Chemotherapy, Guangxi Medical University Cancer Hospital, Nanning, Guangxi, China; University of Navarra School of Medicine and Center for Applied Medical Research (CIMA), SPAIN

## Abstract

The enhancer of zeste homolog 2 (EZH2) plays a critical role in different components of anti-tumor immunity. However, the specific role of EZH2 in modulating MHC Class I antigen presentation and T cell infiltration have not been investigated in HCC. This study analyzed the expression and clinical significance of EZH2 in HCC. The EZH2 genetic alterations were identified using cBioPortal. The EZH2 mRNA and protein levels were found to be significantly higher in HCC than in adjacent normal liver tissues in multiple datasets from the GEO and TCGA databases. High expression of EZH2 was significantly correlated with poor overall survival, disease-specific survival, progression-free survival, and relapse-free survival in almost all patients with HCC. The gene set variance analysis (GSVA) showed that the expression of EZH2 is positively correlated with an immunosuppressive microenvironment and negatively correlated with major MHC class I antigen presentation molecules. Gene set enrichment analysis (GSEA) showed that high EZH2 expression is positively associated with the MYC and glycolysis signaling pathway and negatively associated with the interferon-gamma signaling pathway in HCC tissues. These findings demonstrate that EZH2 is a potential prognostic biomarker and therapeutic target in HCC.

## Introduction

Hepatocellular carcinoma (HCC) is the sixth most prevalent cancer worldwide [1]. Because most patients are diagnosed in advanced stages, the 5-years overall survival rate is only 10% [2]. Current treatment options that include the use of sorafenib and regorafenib provide only marginal benefits for advanced HCC [3, 4]. Immunotherapy with anti-PD-1 antibodies like pembrolizumab has shown a substantial survival benefit in certain cancers, such as Hodgkin’s disease and melanoma [5, 6]. However, the success of immunotherapy has been much more limited in other cancer types, such as HCC [7]. Therefore, there is a need to identify new therapies for the effective treatment of HCC.

The methyltransferase Enhancer of Zeste Homolog 2 (EZH2) is the catalytic subunit of Polycomb Repressor Complex 2 (PRC2), which catalyzes histone H3 methylation on lysine 27 (H3K27). The H3K27me3 histone modification is frequently associated with repressed chromatin conformation and downstream gene expression [8, 9]. Previous studies have reported that EZH2 plays an essential role in anti-tumor immunity. Peng *et al*. reported that treatment with an inhibitor of EZH2 together with an inhibitor of DNA methyltransferase 1 (DNMT1) led to better tumor growth suppression than either approach alone in ovarian tumor-bearing mice [10, 11]. Previous studies have shown that the disruption of EZH2 in regulatory T cells (Tregs) can enhance anti-tumor immunity by diminishing the suppressive activity of Tregs and enhancing T cell infiltration in the tumor [12, 13]. Zingg *et al*. showed that EZH2 inactivation could reverse melanoma resistance mechanisms and synergize with anti-CTLA-4 and IL-2 immunotherapy to suppress melanoma growth [14]. Ennishi *et al*. showed a strong correlation between EHZ2 mutation enrichment and MHC class I and class II expression deficiency in diffuse large B-cell lymphoma [15]. In hepatocellular carcinoma, the inhibition of EZH2 by small-molecule or genetic inhibitors can enhance HCC cell eradication by NK cells in an NK group 2D (NKG2D) ligand-dependent manner [16]. However, the potential relationship between EZH2 and the immune microenvironment in HCC is still unclear.

Furthermore, clinical investigations have shown that EZH2 is aberrantly upregulated in various malignant tumors such as prostate and breast cancer and is associated with advanced stages and poor prognosis [9, 17]. Previous studies have reported that increased expression of EZH2 was frequently detected in HCC tissues and correlated with the aggressiveness and poor prognosis of HCCs [18–21]. However, these results were based on liver tissue samples from a small patient cohort, and the criteria for screening for positive expression of EZH2 were not elaborate. Therefore, further studies are required to demonstrate better the prognostic value of EZH2 in HCC.

This study aimed to analyze the expression and clinical significance of Enhancer of Zeste Homolog 2 in Hepatocellular carcinoma. We comprehensively examined the expression of EZH2, its correlation with prognosis, and the status of different tumor-infiltrating immune cells based on data from patients with HCC in The Cancer Genome Atlas (TCGA) and various public databases. Our results potentially revealed new targets and strategies that could be applied for HCC diagnosis and treatment.

## Materials and methods

### EZH2 gene expression analysis

The EZH2 mRNA expression in the HCC and control samples were analyzed using the GSE10143 [22], GSE14520 [23], GSE36376 [24], GSE54236 [25], GSE64041 [26] and GSE76427 [27] datasets in the GEO database. The expression levels of EZH2 in different datasets were detected. Samples were classified into high-/low-expression groups by the median expression value of EZH2. We also downloaded the normalized gene-level RNA-Seq data and clinical information across 33 cancer types from the UCSC Xena (https://xenabrowser.net/), including LIHC. The expression of EZH2 in 371 primary HCC tissues from patients with different tumor sub-groups was analyzed based on individual cancer stages, tumor grade or other clinicopathological features and tumor stages, and 50 adjacent normal liver tissues at the TCGA database.

### EZH2 protein expression analysis

The expression data of EZH2 protein from HCC and normal liver tissue samples at the TCGA database were obtained from The Human Protein Atlas (http://www.proteinatlas.org) database [28].

### Survival analysis in Kaplan-Meier plotter

The Kaplan-Meier Plotter (http://www.kmplot.com) [29], a robust online publicly available database, was used to study the relationship between the expression of EZH2 and patient clinical outcomes for liver hepatocellular carcinoma. We determined the prognostic value of mRNA expression of EZH2 according to progression-free survival (PFS), relapse-free survival (RFS), disease-specific survival (DSS), and overall survival (OS) in the hepatocellular carcinoma. A total of 370 patients were included in this dataset. Hazard ratios (HRs) with 95% confidence intervals (CI) and log-rank *p*-values were calculated. We also explored the effect of EZH2 expression combined with different clinicopathological features on liver hepatocellular carcinoma. The independent TCGA LIHC data was used to examine the association between the expression of EZH2 and survival.

### TCGA data and tumor-infiltration analysis

Gene sets of 28 subpopulations of tumor-infiltrating lymphocytes were obtained from a previous study [30], including cell types related to adaptive immunity (activated, central memory, effector memory CD4+ and CD8+ T cells, γδ T cells, type 1 helper T (TH1) cells, TH2 cells, TH17 cells, regulatory T cells, follicular helper T cells, and activated, immature and memory B cells), and cell types related to innate immunity (macrophages; monocytes; mast cells; eosinophils; neutrophils; activated, plasmacytoid and immature dendritic cells; natural killer cells; natural killer T cells and myeloid-derived suppressor cell). We used the gene set variance analysis (GSVA) program to calculate the absolute enrichment score of gene signatures for immune cells in each sample, which is referred to as relative immune cell abundance [31]. The Spearman correlation between EZH2 expression level and GSVA score was calculated for each type of immune cell across liver hepatocellular carcinoma, considering FDR < 0.05 as statistical significance.

### Dene set enrichment analysis

To identify the specific gene sets or pathways associated with high EZH2, we performed differential gene expression analysis for LIHC TCGA RNA-Seq data based on EZH2 status. Patients with EZH2 above the median were defined as ‘EZH2-High’ and those with EZH2 below the median defined as ‘EZH2-Low’. Differential expression genes (p-value < 0.01, FDR < 0.05) were ranked by logFC from high to low and then selected for gene set enrichment analysis (GSEA) with gene sets from MSigDB [32].

### TISIDB analysis

The TISIDB database (http://cis.hku.hk/TISIDB) integrates 988 reported immune-related anti-tumor genes, high-throughput screening techniques, molecular profiling, and paracancerous multi-omics data, and various resources for immunological data retrieved from seven public databases [33]. The database enables analyses of correlations for selected genes with lymphocytes, immunomodulators, and chemokines. We employed the TISIDB database to investigate correlations between EZH2 expression and major histocompatibility complex (MHC) molecules.

### c-BioPortal database analysis

The cBio Cancer Genomics Portal (http://cbioportal.org) has multidimensional cancer genomics data sets [34]. Mutation, copy number variation (CNV), and gene co-occurrence of EZH2 in HCC were analyzed using the c-BioPortal tool. The tab OncoPrint displayed an overview of genetic alterations per sample in EZH2.

### Statistical analysis

All the statistical analyses were performed using R software, version 3.6.0 (The R Foundation for Statistical Computing, http://www.r-project.org/). The t-test was used to analyze the variance in expression levels of EZH2 between different groups. P values were determined by unpaired two-tailed Student’s t test. The Survival curves were estimated using the Kaplan-Meier method. The survival analyses were performed by R programming of “survival” and “survminer” packages. The best cut-off values of gene expression were determined by algorithms embedded in the KM plotter. The log-rank test p< 0.05 indicates the significance of survival time differences. The correlations between EZH2 and immune signature score or gene expression levels were calculated using the Spearman method. Spearman correlation analysis using GraphPad Prism. Gene set enrichment analysis was performed by R programming of “GSEABase” and “clusterProfiler” package. Gene set variance analysis was performed by R programming of “GSVA”. The p < 0.05 was considered statistically significant.

## Results

### Elevated mRNA expression levels of EZH2 in HCC

**Fig 1:**
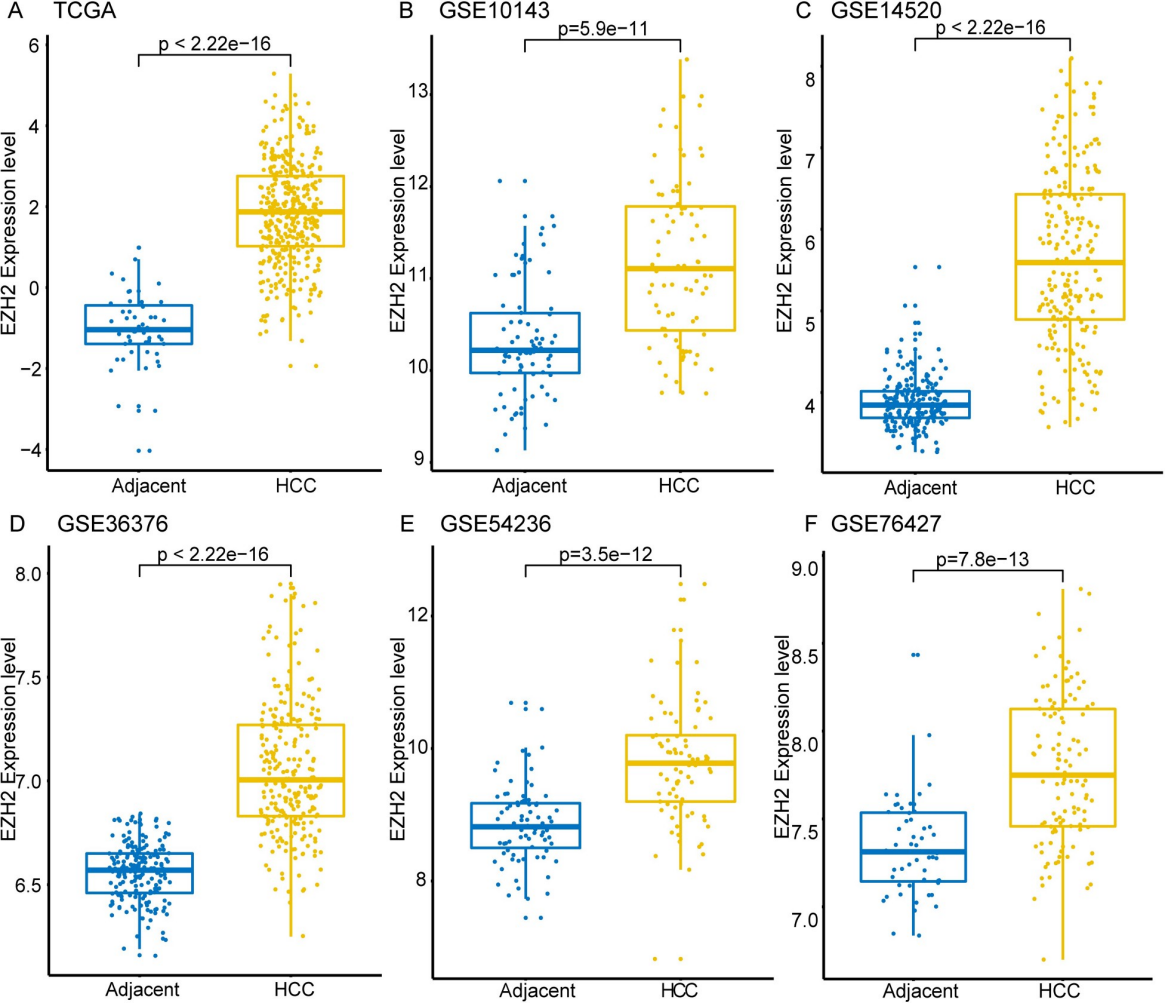
EZH2 transcript levels in HCC and adjacent normal liver tissues. The EZH2 transcript levels in HCC and adjacent normal liver tissues from the (A) TCGA database (371 HCC and 50 normal liver tissues) and (B) GSE10143; (C) GSE14520; (D) GSE36376; (E) GSE54236; and (F) GSE76427 datasets are shown.

We observed significantly higher expression of EZH2 mRNA in the HCC than in the normal liver tissues (Fig 1A) from the TCGA database. Similarly, significantly higher levels of EZH2 mRNA were observed in HCC tissues than in adjacent normal liver tissues from multiple GEO datasets (Fig 1B–1F). These results demonstrated that EZH2 is overexpressed in HCC.

### The expression of EZH2 was positively correlated with the development of HCC

**Fig 2:**
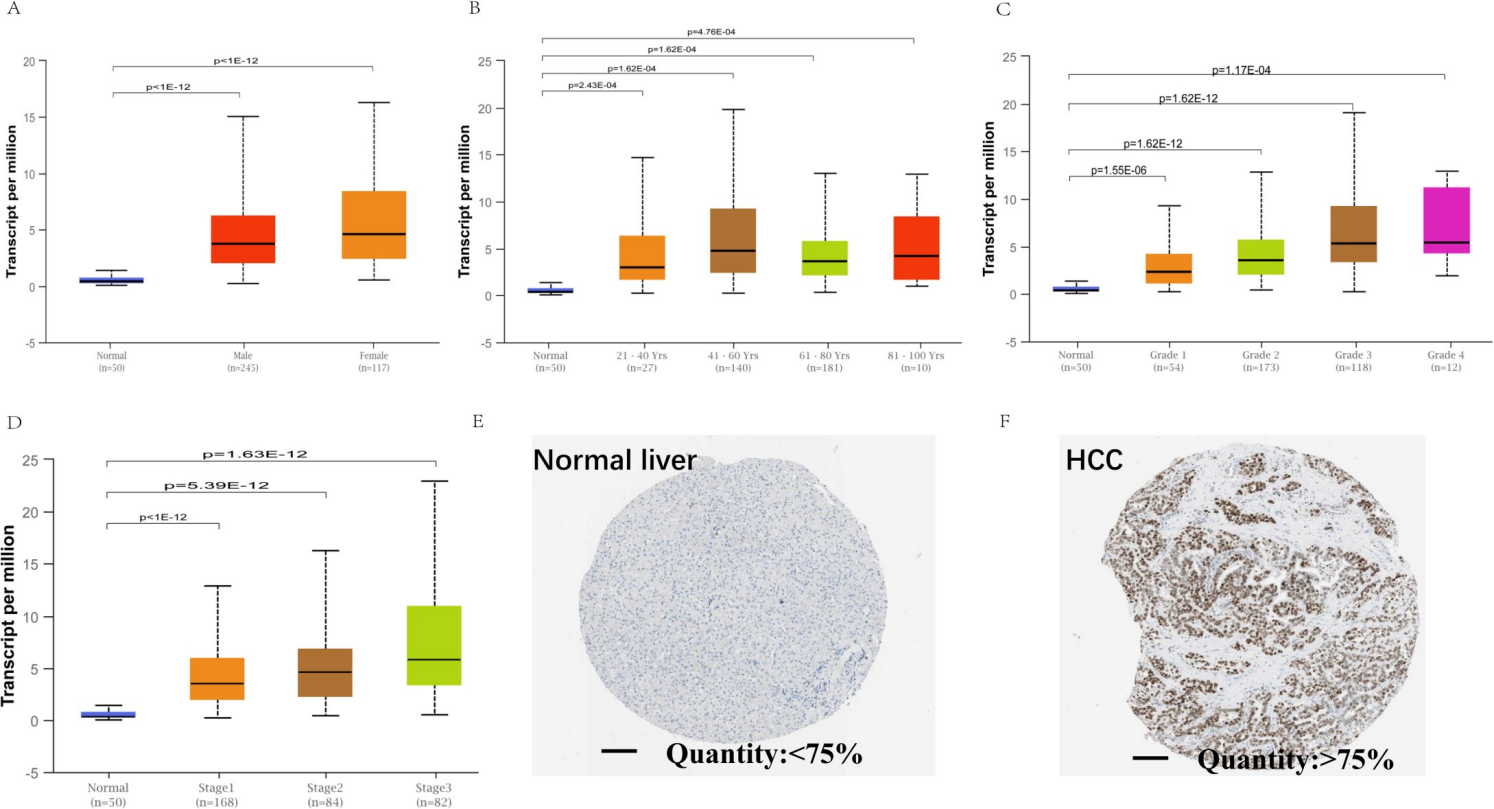
EZH2 mRNA expression in subgroups of patients with HCC, stratified based on gender, age and other criteria and protein expression. (A) The histogram plot shows relative expression of EZH2 in normal individuals of either gender and male or female LIHC patients, respectively. (B) The histogram plot shows relative expression of EZH2 in normal individuals of any age or in LIHC patients aged 21–40, 41–60, 61–80, or 81–100 yr. (C) The histogram plot shows EZH2 mRNA expression in grades 1–4 HCC patients. As shown, EZH2 mRNA expression is incrementally upregulated with increasing tumor grades. (D) The histogram plot shows EZH2 mRNA expression levels in stages 1–3 HCC patients. As shown, EZH2 mRNA levels show incremental upregulation with increasing tumor stages; (E-F) Representative images show EZH2 protein expression in HCC and adjacent normal liver tissues that were obtained from The Human Protein Atlas database. The EZH2 protein expression was analyzed by immunohistochemistry. The scale bar is 200μm.

When the expression of EZH2 in HCC specimens from different subgroups of HCC patients in the TCGA database were analyzed, female and older HCC patients showed a significantly higher expression of EZH2 in their liver tissues than in the normal liver tissues (Fig 2A and 2B), the mRNA levels of EZH2 were found to be significantly increased in the higher tumor grades and stages compared to normal tissues (Fig 2C and 2D). Besides, a significantly higher expression of EZH2 was observed in the early stages and grade 1 of HCC than in the normal liver tissues (Fig 2C and 2D). Furthermore, analysis of the Human Protein Atlas database showed that the EZH2 protein levels in the HCC tissues were 75% higher quantity than in the normal liver tissue samples (Fig 2E and 2F).

### High EZH2 expression is associated with poor prognosis

**Fig 3:**
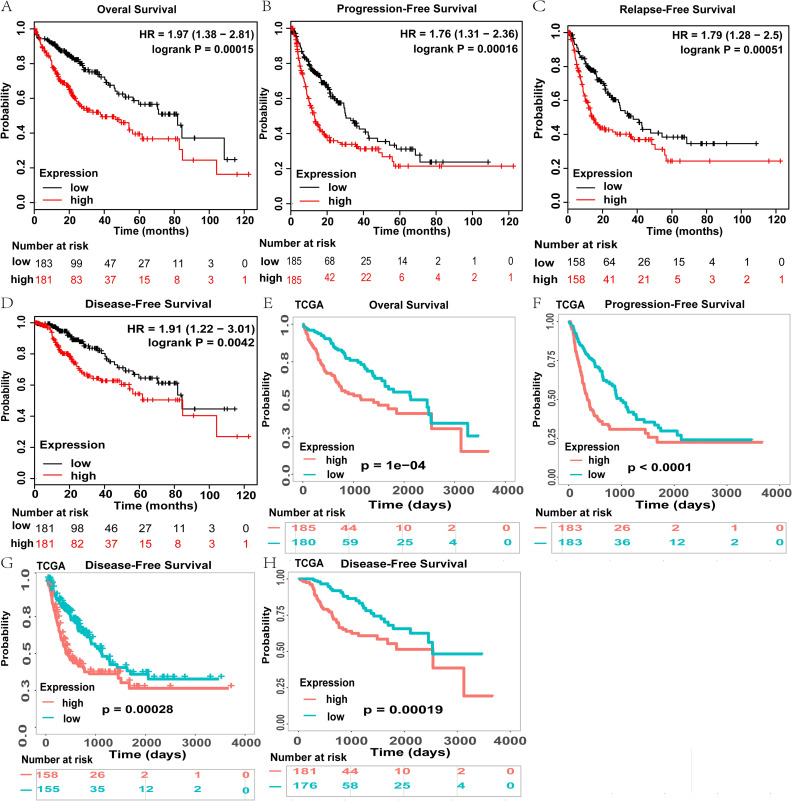
High EZH2 expression predicts poor prognosis in HCC patients. (A–D) High EZH2 expression was correlated with worse OS, PFS, RFS and DSS in Kaplan-Meier plotter database HCC cohort. (E–H) High EZH2 expression was correlated with worse OS, PFS, RFS and DSS in TCGA HCC cohort (n = 364, n = 370, n = 316, n = 362). The numbers below the figures denote the number of patients at risk in each group. The hazard ratio (HR) was calculated based on the Cox Proportional-Hazards model.

The Kaplan Meier (KM) plotter online tool was used to establish the relationship between EZH2 expression and the survival outcomes of HCC cohorts with availed survival information. High expression of EZH2 was associated with poor prognosis in HCC (OS: HR = 1.97, 95% CI = 1.38 to 2.81, p<0.001; PFS: HR = 1.76, 95% CI = 1.31 to 2.36, p<0.001; RFS: HR = 1.79, 95% CI = 1.28 to 2.50, p<0.001; DSS: HR = 1.91, 95% CI = 1.22 to 3.01, p = 0.0042; Fig 3A–3D). In an independent cohort (TCGA-LIHC), HCC with high EZH2 expression had a significantly poorer OS, PFS, DFS, and DSS than those with low expression of EZH2 (Fig 3E–3H).

When the association between EZH2 expression and different clinical characteristics of HCC were investigated, the Kaplan Meier analysis found high expression of EZH2 to correlate with both poor OS and PFS in male (OS: HR = 2.74, p = 3.80E-05; PFS: HR = 2.40, p = 1.10E-05), female (OS: HR = 2.20, p = 0.006; PFS: HR = 2.26, p = 0.0018), Asian (OS: HR = 5.51, p = 3.50E-07; PFS: HR = 3.84, p = 1.20E-05), White (OS: HR = 1.74, p = 0.0178; PFS: HR = 1.84, p = 0.0045), alcohol consuming (OS: HR = 2.42, p = 0.0136; PFS: HR = 3.62, p = 2.40E-05), and non-alcoholic (OS: HR = 2.24, p = 0.0006; PFS: HR = 2.11, p = 0.0002) patients with hepatitis viral infection (OS: HR = 2.98, p = 0.0103; PFS: HR = 2.08, p = 0.0059), and without hepatitis viral infection (OS: HR = 3.04, p = 1.70E-06; PFS: HR = 3.00, p = 4.80E-07) (Table 1).

**Table 1:** Correlation of EZH2 mRNA expression and prognosis in hepatocellular carcinoma with different clinicopathological factors by Kaplan-Meier plotter.

Clinicopathological factors	Overall survival			Progression-free survival		
	N	Hazard ratio	P-value	N	Hazard ratio	P-value
**Gender**						
male	246	2.74(1.66–4.52)	3.80E-05	246	2.40(1.60–3.59)	1.10E-05
female	118	2.20(1.24–3.93)	0.006	120	2.26(1.33–3.84)	0.0018
**Stage**						
1	170	2.15(1.12–4.11)	0.0179	170	2.01(1.20–3.39)	0.0073
2	83	4.54(1.69–12.22)	0.0011	84	1.72(0.90–3.29)	0.0991
1+2	253	2.48(1.49–4.16)	0.0003	254	2.39(1.46–3.92)	0.0004
3	83	2.27(1.26–4.10)	0.0054	83	2.46(1.41–4.30)	0.0012
4	4	-	-	5	-	-
3+4	87	2.10(1.19–3.73)	0.0094	88	2.41(1.40–4.14)	0.001
**Grade**						
1	55	3.40(1.29–8.94)	0.009	55	2.76(1.08–7.06)	0.028
2	174	2.14(1.26–3.66)	0.0042	175	3.29(1.78–6.08)	5.40E-05
3	118	2.72(1.48–5.02)	0.0008	119	2.14(1.29–3.54)	0.0025
4	12	-	-	12	-	-
**AJCC_T**						
1	180	2.11(1.14–3.90)	0.0149	180	2.24(1.26–3.98)	0.0048
2	90	4.64(1.75–12.28)	0.0007	92	1.86(0.99–3.50)	0.0503
3	78	2.24(1.22–4.10)	0.0073	78	2.38(1.33–4.26)	0.0027
4	13	-	-	13	-	-
**Vascular invasion**						
yes	90	2.80(1.22–6.45)	0.0115	91	2.59(1.16–5.79)	0.0158
None	203	1.98(1.19–3.31)	0.0078	204	1.93(1.22–3.05)	0.0045
**Race**						
White	181	1.74(1.09–2.78)	0.0178	183	1.84(1.20–2.83)	0.0045
Asian	155	5.51(2.64–11.51)	3.50E-07	155	3.84(2.01–7.34)	1.20E-05
**Alcohol consumption**						
yes	115	2.42(1.17–4.98)	0.0136	115	3.62(1.91–6.86)	2.40E-05
none	202	2.24(1.39–3.60)	0.0006	204	2.11(1.40–3.17)	0.0002
**Virus hepatitis**						
yes	150	2.98(1.24–7.14)	0.0103	152	2.08(1.22–3.54)	0.0059
None	167	3.04(1.88–4.90)	1.70E-06	167	3.00(1.92–4.69)	4.80E-07

Specifically, high EZH2 mRNA expression correlated with poor OS and PFS in HCC patients belonging to stages 1+2 (OS: HR = 2.48, *p* = 0.0003; PFS: HR = 2.39, p = 0.0004), stages 3+4 (OS: HR = 2.10, p = 0.0094; PFS: HR = 2.41, p = 0.001), grade 1 (OS: HR = 3.40, p = 0.009; PFS: HR = 2.76, p = 0.01), grade 2 (OS: HR = 2.14, p = 0.0042; PFS: HR = 3.29, p = 5.40E-05), grade 3 (OS: HR = 2.72, p = 0.0008; PFS: HR = 2.14, p = 0.0025) patients with vascular invasion (OS: HR = 2.80, p = 0.0115; PFS: HR = 2.59, p = 0.0158), and without vascular invasion (OS: HR = 1.98, p = 0.0078; PFS: HR = 1.93, p = 0.0045) (Table 1). Therefore, from these results the upregulation of EZH2 mRNA is significantly correlated with poor OS and PFS in almost all HCC patients regardless of their clinical characteristics.

### EZH2 correlates with immunosuppression

**Fig 4:**
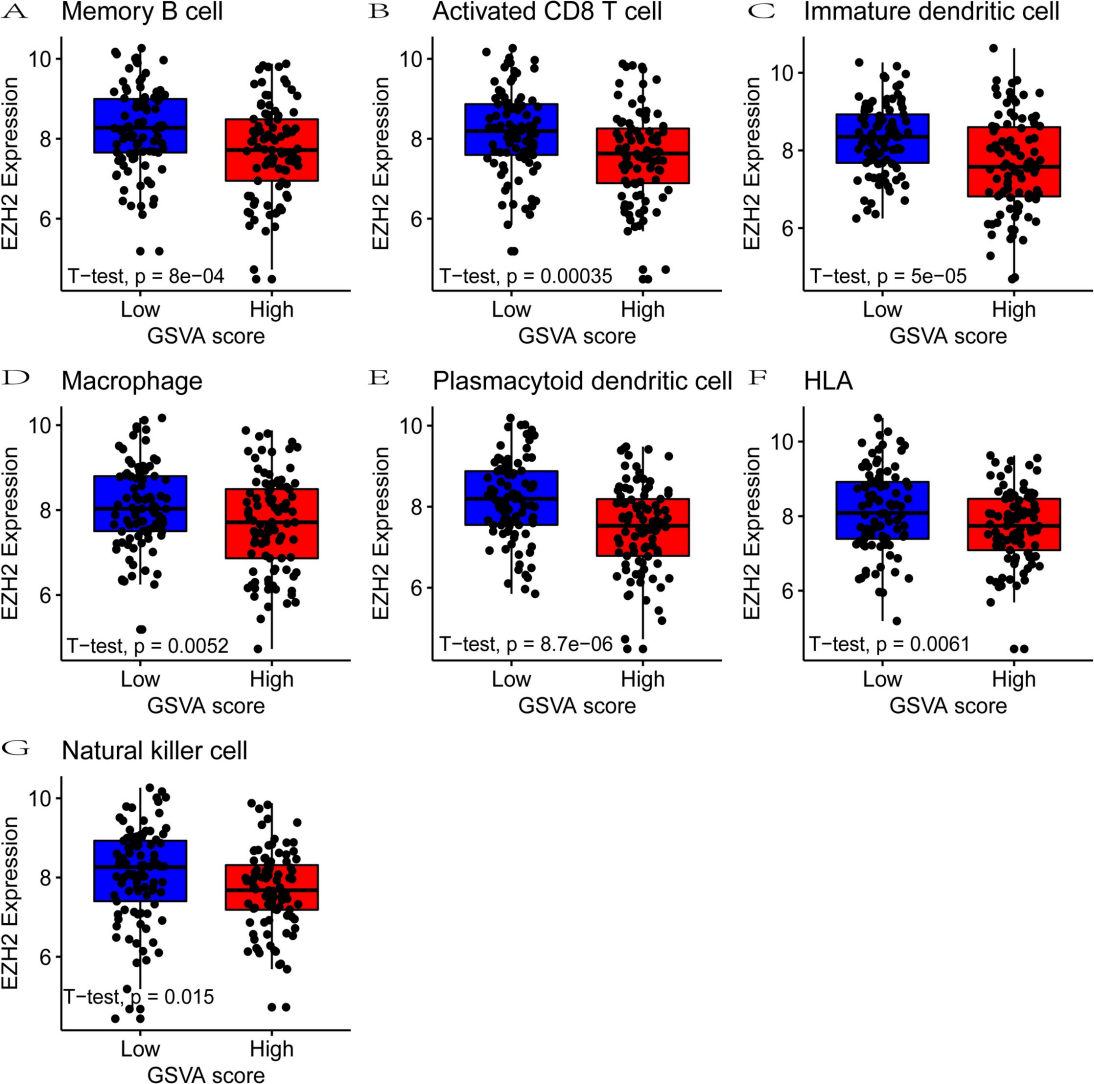
EZH2 is correlated with immune suppressive microenvironment. (A–G) The top 25% of HCC tumors with higher infiltration of activated CD8+ T cells and APCs exhibited substantially reduced EZH2 expression compared with the bottom 25% of HCC tumors. p values were determined by unpaired two-tailed Student’s t test.

**Fig 5:**
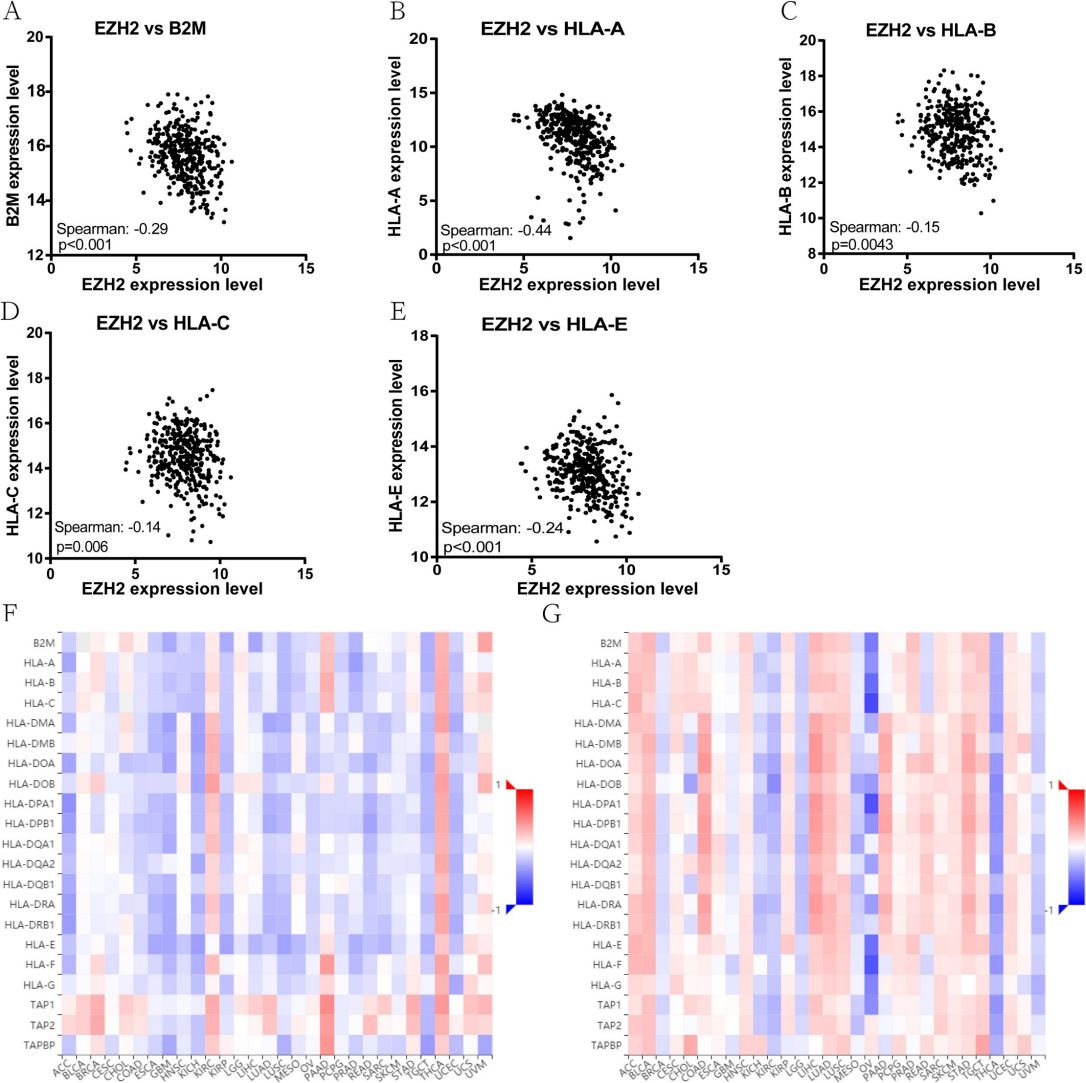
Expression of EZH2 is negatively correlated with major antigen presentation molecules in TCGA HCC data sets. (A-E) Correlation between EZH2 and (A) HLA-A, (B) HLA-B, (C) HLA-C, (D) HLA-E, (E) B2M; (F) Relations between the abundance of MHC molecules and EZH2 expression in TISIDB database; (G) Relations between the abundance of MHC molecules and the methylation of EZH2. Red and blue cells indicate positive and negative correlations, respectively. The color intensity is directly proportional to the strength of the correlations.

The top 25% of HCC tumors with higher infiltration of activated CD8+ T cells and APCs exhibited substantially reduced EZH2 expression compared with the bottom 25% of HCC tumors (Fig 4). The analysis of 371 HCC tumors from TCGA showed a significant inverse correlation between the EZH2 expression levels and major MHC class I antigen presentation molecules, including B2M, HLA-A, HLA-B, HLA-C, and HLA-E (Fig 5A–5E), highlighting a potential regulatory function of EZH2 on antigen presentation in HCC. Furthmore, the expression of EZH2 was negatively correlated with MHC molecules in the TISIDB database. The MHC molecules displaying the negative correlations included B2M (Spearman: ρ = -0.345, p = 9.14e−12), HLA-A (Spearman: ρ = -0.10, p = 0.0791), HLA-B (Spearman: ρ = -0.181, p = 0.00046), HLA-C (Spearman: ρ = -0.166, p = 0.00128), and HLA-E (Spearman: ρ = -0.293, p = 9.03e-09) (Fig 5F). However, the methylation of EZH2 was positively correlated with MHC molecules: B2M (Spearman: ρ = 0.283, p = 3.15e−8), HLA-A (Spearman: ρ = 0.213, p = 3.45e-05), HLA-B (Spearman: ρ = 0.246, p = 1.61e-06), HLA-C (Spearman: ρ = 0.144, p = 0.005), and HLA-E (Spearman: ρ = 0.191, p = 0.0002) (Fig 5G). Thus, the expression of EZH2 is correlated with an immunosuppressive microenvironment.

### Gene set enrichment analyses of EZH2

**Fig 6:**
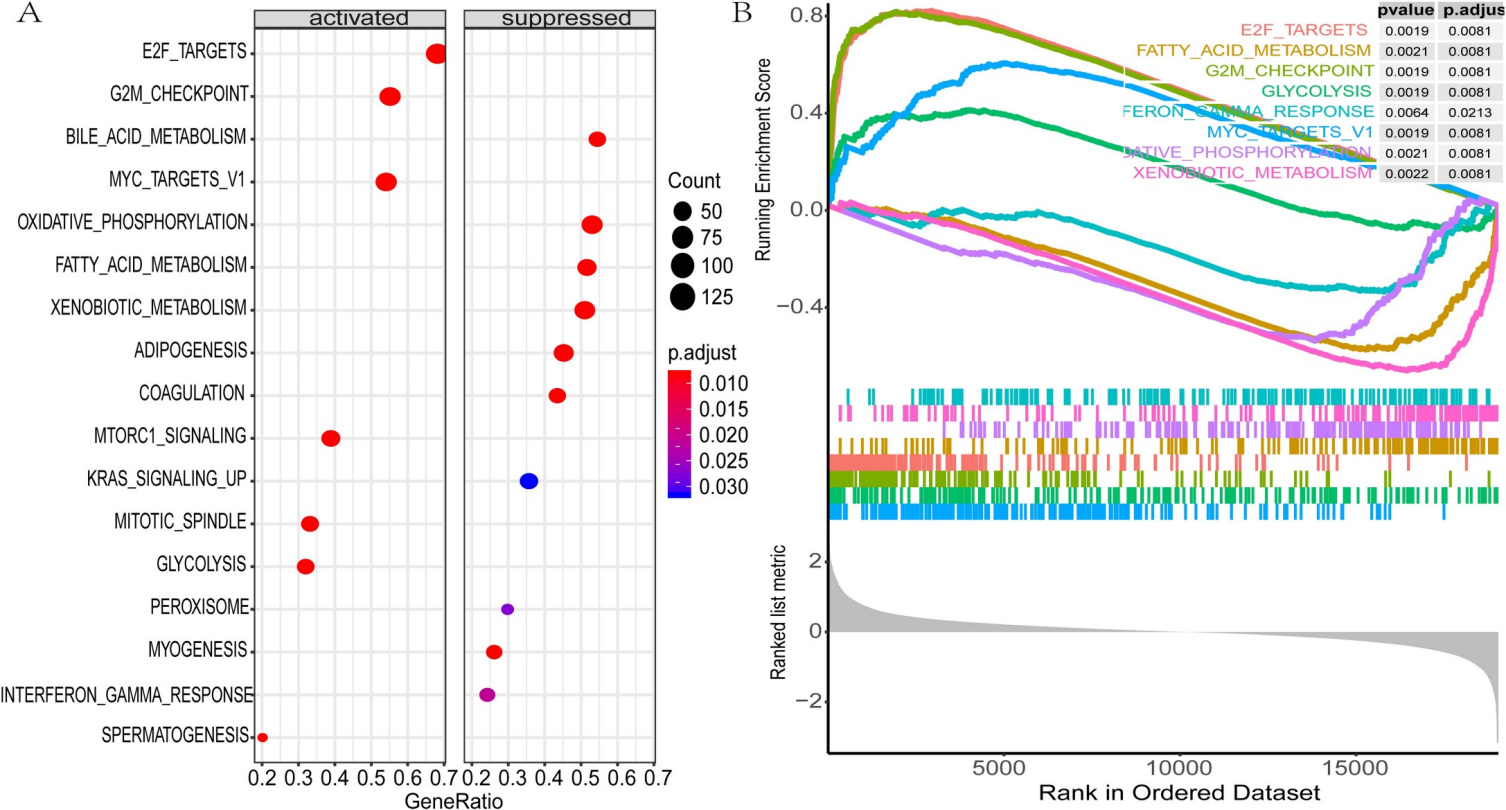
Gene set enrichment analysis analyses of EZH2. (A-B) Gene set enrichment analysis delineates biological pathways and processes correlated with EZH2 expression using gene sets of “h.all.v7.1.symbols” downloaded from the MSigDB database. (A) Activate and suppressed gene sets enriched in patients with high EZH2 expression; (B) Samples were classified into high- and low-EZH2 groups. Each run was performed with 1000 permutations. Enrichment results with significant associations between high- and low-EZH2 groups are shown.

To identify the specific gene signatures associated with EZH2 status, we performed differential gene expression analysis for LIHC TCGA RNA-Seq data based on EZH2 status. Patients with EZH2 above and below the median were defined as ‘EZH2-High’, and ‘EZH2-Low’ patients, respectively. Differential expression genes (p-value < 0.01, FDR < 0.05) were ranked by logFC from high to low and then selected for gene set enrichment analysis (GSEA) with gene sets from MSigDB. From the results of hallmark gene sets, several activated gene signatures (such as MYC_TARGETS_V1, GLYCOLYSIS) and suppressed gene signatures (especially interferon-gamma response) were enriched in LIHC with high EZH2. This showed that high EZH2 expression is positively associated with the MYC and glycolysis signaling pathway and negatively associated with the interferon-gamma signaling pathway (Fig 6A and 6B).

### Genomic alterations of EZH2 in HCC

**Fig 7:**
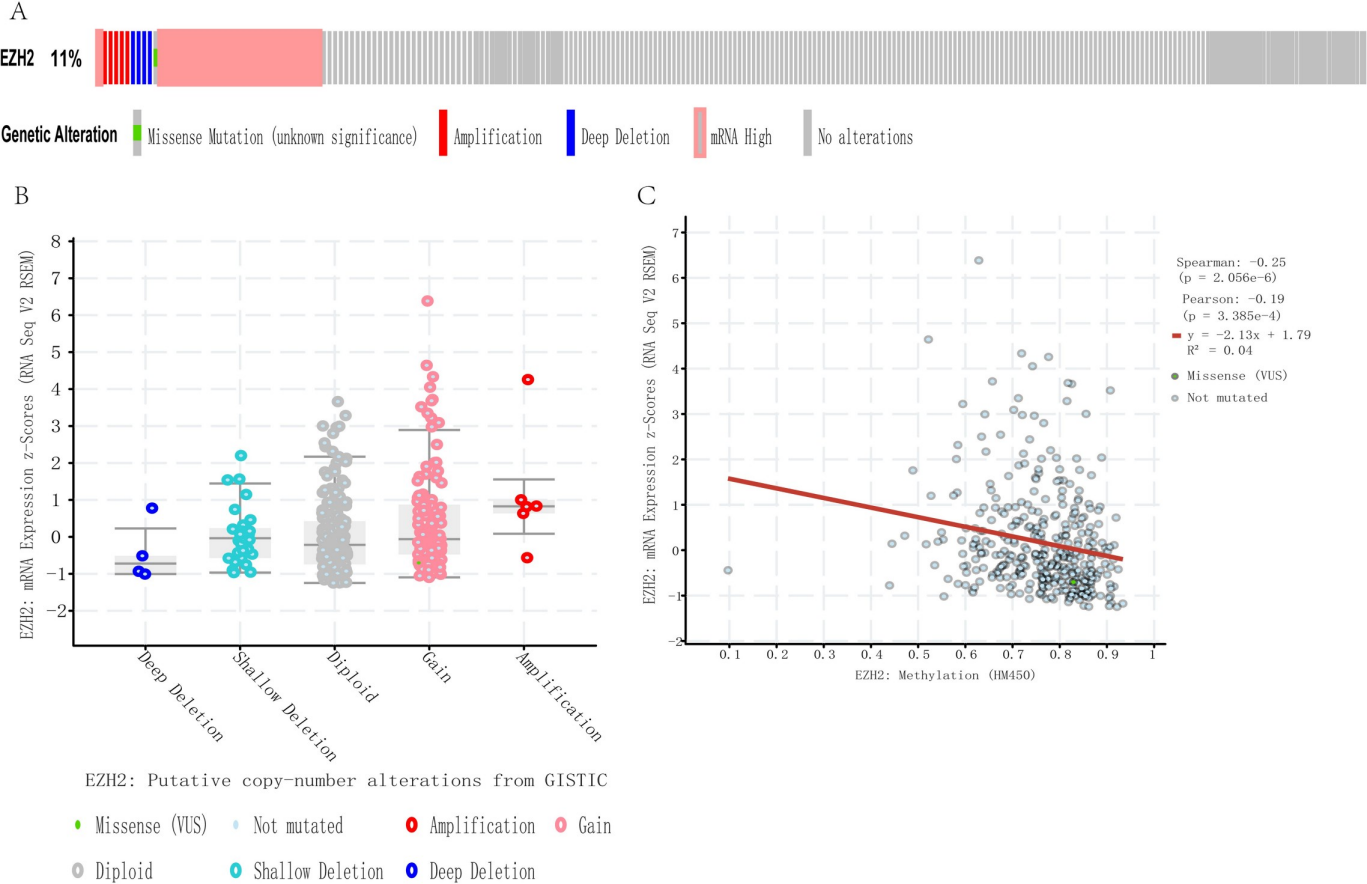
EZH2 genomic alterations in HCC (cBioPortal). OncoPrint of EZH2 alterations in LIHC cohort. The different types of genetic alterations are highlighted in different colors. (B) EZH2 expression in different EZH2 CNV groups. (C) Relations between the methylation of EZH2 and expression level of EZH2.

The analysis of cooperative genomic alterations of EZH2 using cBioPortal for liver hepatocarcinoma (TCGA, provisional) showed that EZH2 was altered in 11% (39 out of 349) of the HCC patients (Fig 7A). These alterations included mRNA upregulation in 8.02% (n = 28), amplification (AMP) in 1.43% (n = 5), mutation in 0.29% (n = 1) and multiple alterations in 0.29% (n = 1) of the cases. Thus, AMP is the most common type of EZH2 CNV in HCC. Amplification of EZH2 results in high expression levels of EZH2 (Fig 7B), whereas methylation of EZH2 results in the low expression levels (Fig 7C).

## Discussion

HCC is one of the most prevalent and fatal forms of liver cancer. The available treatment options are not effective enough and pose life-threatening severe side effects. DNA methylation and modification of histone proteins are well established epigenetic alterations for regulating gene expression. They influence several aspects of cellular physiology and function. Previous studies have demonstrated that overexpression of EZH2 is related to poor prognosis in various cancers [35]. Notably, EZH2 overexpression is reportedly associated with tumor progression and aggressiveness in HCC [21]. Inhibition of EZH2 has been proposed as a therapeutic strategy to induce apoptotic cell death in cancers with a frequent gain of function mutation or over-expression of EZH2 such as melanoma, ovarian cancer, and lymphoma [36–38]. Suresh Bugide *et al*. reported that the genetic and pharmacological inhibition of EZH2 results in the re-expression of several NKG2D ligands that correlates with increased cytotoxicity of NK cells toward HCC cells [16]. These previous studies indicated that overexpression of EZH2 modulates epigenetic silencing of genes involved in tumor progression. Therefore, inhibition of EZH2 could be a potential therapeutic target in HCC.

This study aimed to analyze the expression and clinical significance of Enhancer of Zeste Homolog 2 in Hepatocellular carcinoma. Generally, we found significantly higher EZH2 mRNA levels in HCC than in normal liver tissues. The high expression of EZH2 was significantly related to poor prognosis in multiple cohorts, and poor OS and PFS in almost all HCC patients regardless of their clinical characteristics. The expression of EZH2 was found to correlate with an immunosuppressive microenvironment. The EZH2 expression showed a negative association with major MHC class I antigen presentation molecules. Interestingly, the methylation of EZH2 revealed a positive correlation with major MHC class I antigen presentation molecules. Gene set enrichment analysis showed that high expression of EZH2 is positively associated with the MYC and glycolysis signaling pathway, and negatively associated with the interferon-gamma signaling pathway in HCC tissues. The methylation of EZH2 resulted in the low expression level of EZH2.

We found a positive correlation between the expression of EZH2 and an immunosuppressive microenvironment. The EZH2 expression was negatively associated with an antigen-presenting cell (APC) and CD8+T cell abundance in HCC. Previous studies showed that EZH2 is critical for the recruitment and immunosuppression function of activated regulatory T cells (Tregs) at the sites of inflammation. Moreover, EZH2-deficient Tregs failed to protect mice from developing autoimmunity in a model of naïve T cell-mediated colitis [39, 40]. Our GSEA indicated that high EZH2 expression is positively associated with the MYC and glycolysis signaling pathway and negatively associated with the interferon-gamma signaling pathway. MYC and glycolysis signaling pathway were reportedly associated with tumor progression and aggressiveness [41, 42]. Interferon signaling has been reported to influence the expression of antigen processing and presenting machinery genes [43, 44]. Our study revealed a negative correlation between EZH2 expression and major MHC class I antigen presentation molecules. Therefore, this study has provided a new line of evidence to support the correlation of EZH2 expression with immunosuppression and poor prognosis. Inhibition of EZH2 could be a novel immunotherapeutic target for promoting HCC anti-tumor immunity to overcome checkpoint blockade resistance.

The EZH2 regulated antigen presentation not only in squamous cell carcinomas but also in other tissues. The major histocompatibility complex class I (MHC-I) molecules played a central role in this process by presenting native intracellular proteins or neoantigens produced by cancer cells to effector CD8 + T cells, hence initiating an immune response [45, 46]. Previous studies showed an inverse correlation between EZH2 and MHC class I antigen presentation molecule expression levels in squamous cell carcinomas, such as head, neck, and lung squamous cell carcinoma [47]. Suresh Bugide *et al*. showed that EZH2 inhibition enhances HCC eradication by NK cells and that EZH2 functions partly as an oncogene by inhibiting immune response [16]. Based on these results, we propose that EZH2 inhibition alone or in combination with immune checkpoint inhibitors therapy might be beneficial for HCC patients.

Even though we found important results that can help in the management of HCC, our study had some limitations. The investigations on the clinical significance of EZH2 in HCC were based on data already reported in the Kaplan-Meier plotter. This study failed to verify these outcomes by testing our own clinical samples. Invitro and animal experiments were not conducted to confirm the role of EZH2 in the growth and progression of HCC, and its relationship with the infiltration of immune cells into the tumor microenvironment. Therefore, further studies that can address these limitations are necessary for comprehensive verification of the role played by EZH2 in HCC.

## Conclusions

In conclusion, our results suggest that EZH2 is a potential independent prognostic biomarker for HCC. EZH2 can serve as a biomarker for predicting the outcome of patients with the immunosuppressive microenvironment. Studies using different EZH2 inhibitors and additional HCC preclinical models are needed to confirm our findings.

## Supporting information

S1 File (DOCX)

## References

[1] Heimbach JK, Kulik LM, Finn RS, Sirlin CB, Abecassis MM, Roberts LR, et al AASLD guidelines for the treatment of hepatocellular carcinoma. Hepatology. 2018;67(1):358–80. 10.1002/hep.29086 . 28130846

[2] Cabibbo G, Enea M, Attanasio M, Bruix J, Craxi A, Camma C. A meta-analysis of survival rates of untreated patients in randomized clinical trials of hepatocellular carcinoma. Hepatology. 2010;51(4):1274–83. 10.1002/hep.23485 . 20112254

[3] Bruix J, Qin S, Merle P, Granito A, Huang YH, Bodoky G, et al Regorafenib for patients with hepatocellular carcinoma who progressed on sorafenib treatment (RESORCE): a randomised, double-blind, placebo-controlled, phase 3 trial. Lancet. 2017;389(10064):56–66. 10.1016/S0140-6736(16)32453-9 . 27932229

[4] Llovet JM, Ricci S, Mazzaferro V, Hilgard P, Gane E, Blanc JF, et al Sorafenib in advanced hepatocellular carcinoma. The New England journal of medicine. 2008;359(4):378–90. 10.1056/NEJMoa0708857 . 18650514

[5] Ansell SM, Lesokhin AM, Borrello I, Halwani A, Scott EC, Gutierrez M, et al PD-1 blockade with nivolumab in relapsed or refractory Hodgkin's lymphoma. The New England journal of medicine. 2015;372(4):311–9. 10.1056/NEJMoa1411087 25482239 PMC4348009

[6] Wolchok JD, Chiarion-Sileni V, Gonzalez R, Rutkowski P, Grob JJ, Cowey CL, et al Overall Survival with Combined Nivolumab and Ipilimumab in Advanced Melanoma. The New England journal of medicine. 2017;377(14):1345–56. 10.1056/NEJMoa1709684 28889792 PMC5706778

[7] El-Khoueiry AB, Sangro B, Yau T, Crocenzi TS, Kudo M, Hsu C, et al Nivolumab in patients with advanced hepatocellular carcinoma (CheckMate 040): an open-label, non-comparative, phase 1/2 dose escalation and expansion trial. Lancet. 2017;389(10088):2492–502. 10.1016/S0140-6736(17)31046-2 . 28434648 PMC7539326

[8] Cao R, Wang L, Wang H, Xia L, Erdjument-Bromage H, Tempst P, et al Role of histone H3 lysine 27 methylation in Polycomb-group silencing. Science. 2002;298(5595):1039–43. 10.1126/science.1076997 . 12351676

[9] Kim KH, Roberts CW. Targeting EZH2 in cancer. Nature medicine. 2016;22(2):128–34. 10.1038/nm.4036 26845405 PMC4918227

[10] Peng D, Kryczek I, Nagarsheth N, Zhao L, Wei S, Wang W, et al Epigenetic silencing of TH1-type chemokines shapes tumour immunity and immunotherapy. Nature. 2015;527(7577):249–53. 10.1038/nature15520 26503055 PMC4779053

[11] Kugelberg E. Tumour immunology: Reducing silence to improve therapy. Nature reviews Immunology. 2015;15(12):730 10.1038/nri3941 . 26542634

[12] Wang D, Quiros J, Mahuron K, Pai CC, Ranzani V, Young A, et al Targeting EZH2 Reprograms Intratumoral Regulatory T Cells to Enhance Cancer Immunity. Cell reports. 2018;23(11):3262–74. 10.1016/j.celrep.2018.05.050 29898397 PMC6094952

[13] Goswami S, Apostolou I, Zhang J, Skepner J, Anandhan S, Zhang X, et al Modulation of EZH2 expression in T cells improves efficacy of anti-CTLA-4 therapy. The Journal of clinical investigation. 2018;128(9):3813–8. 10.1172/JCI99760 29905573 PMC6118570

[14] Zingg D, Arenas-Ramirez N, Sahin D, Rosalia RA, Antunes AT, Haeusel J, et al The Histone Methyltransferase Ezh2 Controls Mechanisms of Adaptive Resistance to Tumor Immunotherapy. Cell reports. 2017;20(4):854–67. 10.1016/j.celrep.2017.07.007 . 28746871

[15] Ennishi D, Takata K, Beguelin W, Duns G, Mottok A, Farinha P, et al Molecular and Genetic Characterization of MHC Deficiency Identifies EZH2 as Therapeutic Target for Enhancing Immune Recognition. Cancer discovery. 2019;9(4):546–63. 10.1158/2159-8290.CD-18-1090 . 30705065

[16] Bugide S, Green MR, Wajapeyee N. Inhibition of Enhancer of zeste homolog 2 (EZH2) induces natural killer cell-mediated eradication of hepatocellular carcinoma cells. Proceedings of the National Academy of Sciences of the United States of America. 2018;115(15):E3509–E18. 10.1073/pnas.1802691115 29581297 PMC5899497

[17] Varambally S, Dhanasekaran SM, Zhou M, Barrette TR, Kumar-Sinha C, Sanda MG, et al The polycomb group protein EZH2 is involved in progression of prostate cancer. Nature. 2002;419(6907):624–9. 10.1038/nature01075 . 12374981

[18] Cai MY, Tong ZT, Zheng F, Liao YJ, Wang Y, Rao HL, et al EZH2 protein: a promising immunomarker for the detection of hepatocellular carcinomas in liver needle biopsies. Gut. 2011;60(7):967–76. 10.1136/gut.2010.231993 . 21330577

[19] Cui S, Sun Y, Liu Y, Liu C, Wang J, Hao G, et al MicroRNA137 has a suppressive role in liver cancer via targeting EZH2. Molecular medicine reports. 2017;16(6):9494–502. 10.3892/mmr.2017.7828 29152663 PMC5780008

[20] Sudo T, Utsunomiya T, Mimori K, Nagahara H, Ogawa K, Inoue H, et al Clinicopathological significance of EZH2 mRNA expression in patients with hepatocellular carcinoma. British journal of cancer. 2005;92(9):1754–8. 10.1038/sj.bjc.6602531 15856046 PMC2362028

[21] Sasaki M, Ikeda H, Itatsu K, Yamaguchi J, Sawada S, Minato H, et al The overexpression of polycomb group proteins Bmi1 and EZH2 is associated with the progression and aggressive biological behavior of hepatocellular carcinoma. Laboratory investigation; a journal of technical methods and pathology. 2008;88(8):873–82. 10.1038/labinvest.2008.52 . 18591938

[22] Hoshida Y, Villanueva A, Kobayashi M, Peix J, Chiang DY, Camargo A, et al Gene expression in fixed tissues and outcome in hepatocellular carcinoma. The New England journal of medicine. 2008;359(19):1995–2004. 10.1056/NEJMoa0804525 18923165 PMC2963075

[23] Roessler S, Jia HL, Budhu A, Forgues M, Ye QH, Lee JS, et al A unique metastasis gene signature enables prediction of tumor relapse in early-stage hepatocellular carcinoma patients. Cancer research. 2010;70(24):10202–12. 10.1158/0008-5472.CAN-10-2607 21159642 PMC3064515

[24] Lim HY, Sohn I, Deng S, Lee J, Jung SH, Mao M, et al Prediction of disease-free survival in hepatocellular carcinoma by gene expression profiling. Annals of surgical oncology. 2013;20(12):3747–53. 10.1245/s10434-013-3070-y . 23800896

[25] Villa E, Critelli R, Lei B, Marzocchi G, Camma C, Giannelli G, et al Neoangiogenesis-related genes are hallmarks of fast-growing hepatocellular carcinomas and worst survival. Results from a prospective study. Gut. 2016;65(5):861–9. 10.1136/gutjnl-2014-308483 . 25666192

[26] Makowska Z, Boldanova T, Adametz D, Quagliata L, Vogt JE, Dill MT, et al Gene expression analysis of biopsy samples reveals critical limitations of transcriptome-based molecular classifications of hepatocellular carcinoma. The journal of pathology Clinical research. 2016;2(2):80–92. 10.1002/cjp2.37 27499918 PMC4907058

[27] Grinchuk OV, Yenamandra SP, Iyer R, Singh M, Lee HK, Lim KH, et al Tumor-adjacent tissue co-expression profile analysis reveals pro-oncogenic ribosomal gene signature for prognosis of resectable hepatocellular carcinoma. Molecular oncology. 2018;12(1):89–113. 10.1002/1878-0261.12153 29117471 PMC5748488

[28] Uhlen M, Fagerberg L, Hallstrom BM, Lindskog C, Oksvold P, Mardinoglu A, et al Proteomics. Tissue-based map of the human proteome. Science. 2015;347(6220):1260419 10.1126/science.1260419 . 25613900

[29] Nagy A, Lanczky A, Menyhart O, Gyorffy B. Validation of miRNA prognostic power in hepatocellular carcinoma using expression data of independent datasets. Scientific reports. 2018;8(1):9227 10.1038/s41598-018-27521-y 29907753 PMC6003936

[30] Charoentong P, Finotello F, Angelova M, Mayer C, Efremova M, Rieder D, et al Pan-cancer Immunogenomic Analyses Reveal Genotype-Immunophenotype Relationships and Predictors of Response to Checkpoint Blockade. Cell reports. 2017;18(1):248–62. 10.1016/j.celrep.2016.12.019 . 28052254

[31] Hanzelmann S, Castelo R, Guinney J. GSVA: gene set variation analysis for microarray and RNA-seq data. BMC bioinformatics. 2013;14:7 10.1186/1471-2105-14-7 23323831 PMC3618321

[32] Subramanian A, Tamayo P, Mootha VK, Mukherjee S, Ebert BL, Gillette MA, et al Gene set enrichment analysis: a knowledge-based approach for interpreting genome-wide expression profiles. Proceedings of the National Academy of Sciences of the United States of America. 2005;102(43):15545–50. 10.1073/pnas.0506580102 16199517 PMC1239896

[33] Ru B, Wong CN, Tong Y, Zhong JY, Zhong SSW, Wu WC, et al TISIDB: an integrated repository portal for tumor-immune system interactions. Bioinformatics. 2019;35(20):4200–2. 10.1093/bioinformatics/btz210 . 30903160

[34] Gao J, Aksoy BA, Dogrusoz U, Dresdner G, Gross B, Sumer SO, et al Integrative analysis of complex cancer genomics and clinical profiles using the cBioPortal. Science signaling. 2013;6(269):pl1 10.1126/scisignal.2004088 23550210 PMC4160307

[35] Gan L, Yang Y, Li Q, Feng Y, Liu T, Guo W. Epigenetic regulation of cancer progression by EZH2: from biological insights to therapeutic potential. Biomarker research. 2018;6:10 10.1186/s40364-018-0122-2 29556394 PMC5845366

[36] Zingg D, Debbache J, Schaefer SM, Tuncer E, Frommel SC, Cheng P, et al The epigenetic modifier EZH2 controls melanoma growth and metastasis through silencing of distinct tumour suppressors. Nature communications. 2015;6:6051 10.1038/ncomms7051 . 25609585

[37] Jones BA, Varambally S, Arend RC. Histone Methyltransferase EZH2: A Therapeutic Target for Ovarian Cancer. Molecular cancer therapeutics. 2018;17(3):591–602. 10.1158/1535-7163.MCT-17-0437 29726819 PMC5939583

[38] Lue JK, Amengual JE. Emerging EZH2 Inhibitors and Their Application in Lymphoma. Current hematologic malignancy reports. 2018;13(5):369–82. 10.1007/s11899-018-0466-6 . 30112706

[39] DuPage M, Chopra G, Quiros J, Rosenthal WL, Morar MM, Holohan D, et al The chromatin-modifying enzyme Ezh2 is critical for the maintenance of regulatory T cell identity after activation. Immunity. 2015;42(2):227–38. 10.1016/j.immuni.2015.01.007 25680271 PMC4347854

[40] Yang XP, Jiang K, Hirahara K, Vahedi G, Afzali B, Sciume G, et al EZH2 is crucial for both differentiation of regulatory T cells and T effector cell expansion. Scientific reports. 2015;5:10643 10.1038/srep10643 26090605 PMC4473539

[41] Stine ZE, Walton ZE, Altman BJ, Hsieh AL, Dang CV. MYC, Metabolism, and Cancer. Cancer discovery. 2015;5(10):1024–39. 10.1158/2159-8290.CD-15-0507 26382145 PMC4592441

[42] Jiang Z, Liu Z, Li M, Chen C, Wang X. Increased glycolysis correlates with elevated immune activity in tumor immune microenvironment. EBioMedicine. 2019;42:431–42. 10.1016/j.ebiom.2019.03.068 30935888 PMC6491961

[43] Beatty GL, Paterson Y. Regulation of tumor growth by IFN-gamma in cancer immunotherapy. Immunologic research. 2001;24(2):201–10. 10.1385/IR:24:2:201 . 11594457

[44] Ikeda H, Old LJ, Schreiber RD. The roles of IFN gamma in protection against tumor development and cancer immunoediting. Cytokine & growth factor reviews. 2002;13(2):95–109. 10.1016/s1359-6101(01)00038-7 . 11900986

[45] Kosaloglu-Yalcin Z, Lanka M, Frentzen A, Logandha Ramamoorthy Premlal A, Sidney J, Vaughan K, et al Predicting T cell recognition of MHC class I restricted neoepitopes. Oncoimmunology. 2018;7(11):e1492508 10.1080/2162402X.2018.1492508 30377561 PMC6204999

[46] Leone P, Shin EC, Perosa F, Vacca A, Dammacco F, Racanelli V. MHC class I antigen processing and presenting machinery: organization, function, and defects in tumor cells. Journal of the National Cancer Institute. 2013;105(16):1172–87. 10.1093/jnci/djt184 . 23852952

[47] Zhou L, Mudianto T, Ma X, Riley R, Uppaluri R. Targeting EZH2 Enhances Antigen Presentation, Antitumor Immunity, and Circumvents Anti-PD-1 Resistance in Head and Neck Cancer. Clinical cancer research: an official journal of the American Association for Cancer Research. 2020;26(1):290–300. 10.1158/1078-0432.CCR-19-1351 31562203 PMC6942613

