# Supplementary material for: EZH2 is a negative prognostic biomarker associated with immunosuppression in hepatocellular carcinoma
Source: PLoS One. 2020 Nov 12;15(11):e0242191. doi: 10.1371/journal.pone.0242191 (PMC7660515; doi:10.1371/journal.pone.0242191)
Supplement: S1 File — (DOCX) [file pone.0242191.s001.docx]

All data supporting the findings of the current study are from publicly available databases (GEO: https://www.ncbi.nlm.nih.gov/geo/; TCGA：https://xenabrowser.net/) and online publicly available databases (such as The Human Protein Atlas: http://www. proteinatlas.org; The Kaplan-Meier Plotter :http://www.kmplot.com; The TISIDB database: http://cis.hku.hk/TISIDB; and The cBio Cancer Genomics Portal :http://cbioportal.org).

The GEO access data include: GSE10143, GSE14520, GSE36376, GSE54236, GSE64041 and GSE76427.

We downloaded the normalized gene-level RNA-Seq data and clinical information across 33 cancer types from the UCSC Xena (<https://xenabrowser.net/>).
